# Emergency medicine in Zanzibar: the effect of system changes in the emergency department

**DOI:** 10.1186/s12245-015-0072-5

**Published:** 2015-07-14

**Authors:** Oyvind Thomassen, Clifford Mann, Juma Salum Mbwana, Guttorm Brattebo

**Affiliations:** Department of Anaesthesiology and Intensive care, Haukeland University Hospital, Bergen, Norway; Emergency Department Musgrove Park Hospital, Taunton, England UK; Outpatient Department, Mnazi Mmoja Hospital, Zanzibar, Tanzania

**Keywords:** Emergency medicine, Emergency department, Low-income countries, Quality improvement

## Abstract

**Background:**

Mnazi Mmoja Hospital is a tertiary hospital in Zanzibar serving a population of 1.2 million. The emergency department was overcrowded and understaffed and the hospital management initiated a quality improvement project. The aim of this article is to describe the approach, methods and main results of this quality improvement process.

**Methods:**

The Plan-Do-Study-Act (PDSA) method was used in a five-circle process. In addition, a consensus-based approach was performed to identify areas of improvement.

**Results:**

Over a period of 6 months, regular staff meetings were implemented, a registration system was developed and implemented, the numbers of patients with simple problems were reduced, a simple triage tool was developed and implemented and an emergency room was established.

**Conclusions:**

Change and improvement in health care are achievable despite limited financial resources if a comprehensive, robust and simple system is used. Involvement of all stakeholders from the start, identification and use of change agents, regular feedback and a focus on human resources rather than equipment have been key factors for the success of this project.

## Background

Zanzibar lies 25 miles off the East African coast. It is an archipelago consisting of the two main islands, Unguja and Pemba, covering an area of 650 mile^2^ with a population of 1.3 million. Zanzibar is a semi-autonomous part of Tanzania with a local government, president and some ministries, including the Ministry of Health and Social Welfare. Zanzibar is one of the poorest countries in sub-Saharan Africa [1]. The average annual income is US$ 250, and 55 % of the rural population lives in poverty. One third of the population has not received any education. HIV prevalence is less than 1 %, and life expectancy is 57 years [2]. The child mortality rate is 73 out of 1000 live births which is lower than the mainland. The government’s share of health care expenditure is low, making Zanzibar health care highly dependent on foreign donations [3].

The public health care system is divided into three levels (primary, secondary and tertiary). There are more than 100 primary health care centres on Unguja and Pemba, one secondary hospital at Pemba and two smaller hospitals on Unguja. The only tertiary hospital is Mnazi Mmoja Hospital (MMH), located in the capital Stone Town. MMH has approximately 400 beds and is staffed with doctors, clinical officers and nurses. The specialty departments include paediatrics, neonatal medicine, surgery, internal medicine, physiotherapy, obstetrics, gynaecology, ear nose and throat, ophthalmology and an emergency department. There are no ambulance services on the island.

The emergency department (ED) is today a part of the outpatient department (OPD) and serves a population of 1.2 million and provides 24/7 services. Prior to 2012, the ED did not exist and no personnel were trained or equipped to treat patients in need of emergency care. The patients in need of immediate care had to wait in the long line in the OPD.

During the project period, the OPD staffing consisted of one medical doctor, one assistant medical office (AMO), 12 clinical officers (COs) and 12 nurses. A clinical officer is a mid-level practitioner with 3 years training licensed to work independently. An assistant medical officer is a CO with additional 2 years training. During the daytime, there were four to six COs and six nurses on call and, during the evening and night shift, two COs and two nurses. The medical doctor and the AMO mainly organised the department while the COs performed the consultations. In the OPD, the examination rooms were simply equipped with only a table, a stretcher and a blood pressure device. There was no registration of activity. Some laboratory and radiology services were available in other parts of the hospital. Medications and consumables were available for purchase at a drugstore nearby. Family or friends were responsible for transportation to the hospital, and patients could be admitted after examination.

MMH and Haukeland University Hospital (HUH), Norway, have a long-term cooperation with bilateral exchange of health care workers. During the 2011 ferry disaster claiming more than 2000 lives, personnel from HUH were asked to assist in the OPD. After this request, the management of MMH asked for a long-term commitment from HUH aimed at improving the emergency medical system in the OPD. At the same time, a medical team from England was also asked to assist and joined the project. The objective of this article is to describe the approach, methods and main results of this quality improvement process.

## Methods

One basic theorem in quality improvement is that change is a prerequisite for improvement to happen but that every change does not guarantee improvement. Therefore, it is very important to carefully select the changes, plan them, measure the changes and decide whether this represents an improvement or not [4].

**Fig. 1:**
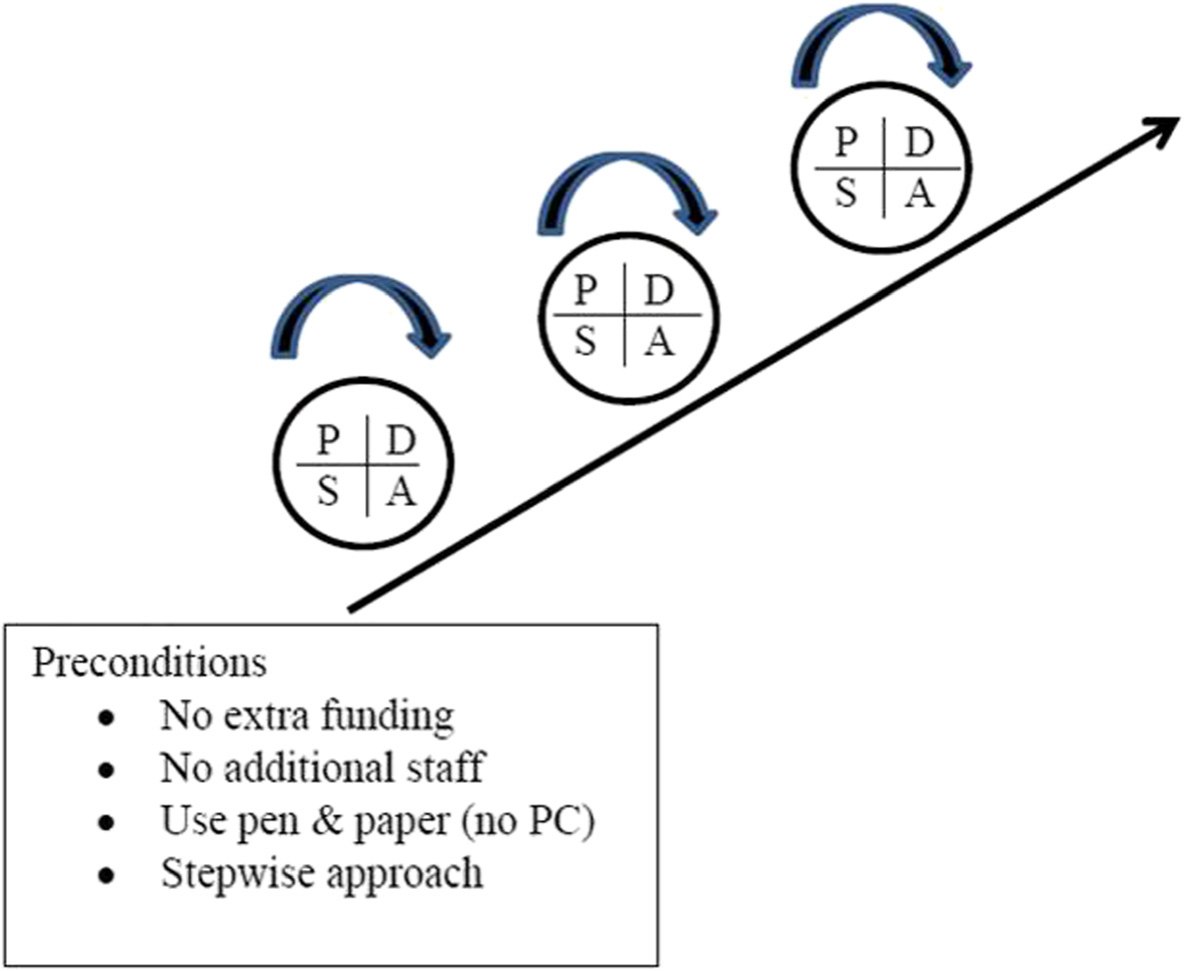
The rapid sequence PDSA model for quality improvement

As HUH had experience in using the PDSA improvement method, this was chosen as the approach. PDSA is the acronym for Plan-Do-Study-Act and describes the stepwise approach of this method [5]. By applying several rapid cycles in succession, substantial improvement can be achieved. Prior to the project (October 2012–April 2013), the following preconditions were agreed: no extra funds, no additional staff, a stepwise approach and only low-tech equipment. A shared and common understanding of these preconditions was a key to a constructive and nurturing process, promoting cooperation with the staff and the management (Fig. 1).

The most important single factor was that there would be no extra funding (OT and GB from Norway and CM with his team from England had their salaries paid by their respective hospitals).

The possible areas for improvement were identified using a consensus-based approach among all OPD personnel. They also agreed on which areas that should be prioritized as shown in Table 1. After this, we decided on five cycles: medical meetings, documentation and registration of patients in the OPD, separate pathway for patients with simple medical conditions, triage for identifying patients with life-threatening conditions and establishing a functioning emergency room with equipment and treatment protocols.

**Table 1:** Areas in most need for quality improvement identified by personnel in OPD in prioritized order

• Load of patients must be lower	
• Establish an emergency room	
• Triage system	
• More equipment	
• Protocols	
• Continue with staff meetings	
• Available medication for life-threatening conditions	
• Education of staff	
• Increase the number of staff	
• Diagnosis, definitions	

## Results

### Cycle 1: implementation of regular staff meetings to discuss areas of quality improvement

The plan for cycle 1 was to implement weekly staff meetings to discuss the quality improvement process. The Zanzibarian “on-time culture” was a challenge. We solved this by starting every meeting with the superior’s weekly messages followed by a teaching session. These two themes were both needed and wanted and encouraged the staff to come on time. The head of department and the head nurse were always on time. In addition, we also introduced regular continuous medical education sessions with lectures, simulation exercises and hands-on, shoulder-to-shoulder skills training as the latter part of these meetings. The staff meetings were carried out every week and became the main meeting point for discussion and exchange of ideas. The personnel that had been on night call (two COs and two nurses) did not attend the meetings. Close to 90 % of the remaining staff attended every week.

### Cycle 2: registration of all OPD patients

The OPD was constantly overcrowded. On any given day, more than a hundred patients were occupying the narrow and dark waiting area. It was obvious that any improvement was almost impossible if the ratio of staff to number of patients remained unchanged. The staff agreed upon that the next plan had to be a registration system in order to explore how many patients each CO had to consult on their shift and which medical problems they presented with.

**Fig. 2:**
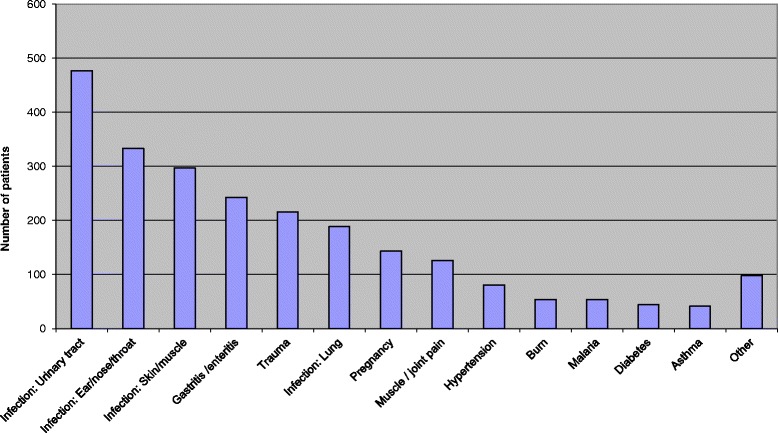
Distribution of medical problem/symptoms during the two week registration period

To avoid registration fatigue, plans were made to prevent increased workload on the already overstretched staff; these included a maximum of 10 s to register one patient and only six key points with direct relevance to be registered: (1) patient, number; (2) age; (3) gender; (4) address; (5) discharged to/admitted to; and (6) diagnosis/problem. The head of department did daily rounds and encouraged staff in order to keep spirits high. The registration forms were collected every morning and the results displayed on a chart on the wall in the OPD. The day-to-day updated summaries increased the motivation to continue registration. During the 2 weeks, a total of 3840 patients were registered. Categories of the medical problems that presented are shown in Fig. 2. The plan was to do registration for 2 weeks but was continued due to feedback from the nurses and clinical officers. The Ministry of Health and Social Welfare had been aware of the long-term understaffing situation, and an additional two COs and one MO were employed in the OPD.

### Cycle 3: reducing the number of patients with simple problems presenting in the OPD

The registration revealed two main findings. Firstly, the total number was three times higher than the hospital administration’s previous estimates (forcing the mean consultation time to be less than 7 min, 24/7). The staff were exhausted, and the examination rooms were overcrowded. Secondly, the top three diagnosis groups did not need emergency care at a hospital. The next plan was to reduce the number of patients with simple problems to generate space and time for those with serious or life-threatening conditions. A reduction of patients would also facilitate better overview of the waiting area and make triage possible.

**Fig. 3:**
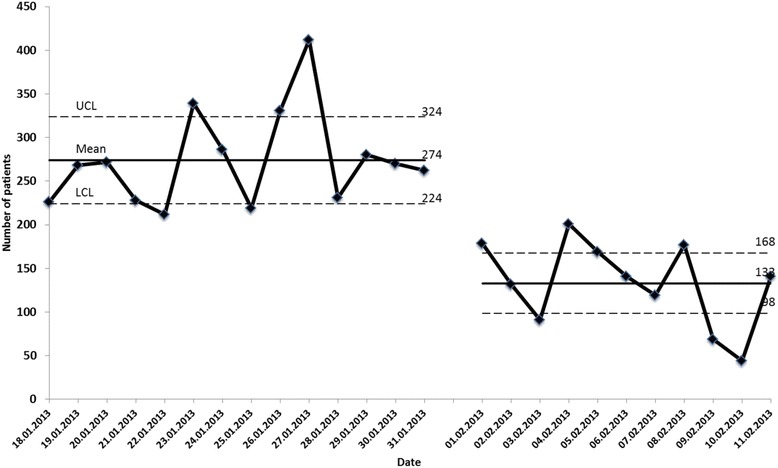
Number of patients in the OPD before and after the opening of the primary health care centre

To achieve this, a close cooperation was established with the nearby primary health care centre at Rahaleo Primary Care Clinic. The OPD staff helped to organize and prepare this primary health care centre to increase its capacity. The head of department broadcast a radio bulletin informing the public of the system change. In addition, staff from the OPD frequently undertook information rounds in the waiting area telling patients with simple problems to go to the primary care service. For patients who could read, written information was posted on the OPD walls. The mean number of patients per day in the OPD was 274 before and 133 after introduction of self-triage (Fig. 3). The number of admissions from Rahaleo to MMH (under triage) was 0–5 daily, indicating that the system functioned well. None of these admissions were for life-threatening conditions.

### Cycle 4: implement a triage system to identify the patients in immediate need of care

At this stage, we had developed the first “self-triage” system and the number of patients had been reduced by approximately 50 %. The next plan was to introduce a system for identifying the patients in need of immediate care. We scrutinized some relevant validated triage systems, but due to poorly developed patient flow between departments, understaffing and underfunding, we concluded that a very simple system with only two categories would be feasible: “red” (needing emergency room care) and “green” (safe to manage in the OPD). During staff meetings, we discussed the appropriate location for triage and inclusion criteria for “red” cases and decided which staff should perform the triage. Table 2 lists the inclusion criteria for “red” cases that were agreed upon.

**Table 2:** Inclusion criteria for code “red”

• Apnoea or severe breathing problems	
• Severe haemorrhage	
• Convulsions	
• Impaired consciousness	
• Open fractures in large bones	
• Severe burns	

The OPD waiting area had six entrances, and due to lack of staff, it was not possible to have a single registration point upon entering the OPD. Therefore, the solution was that all personnel should be constantly aware of the red inclusion criteria, and everyone who spotted a patient who met the criteria should take immediate action. We realized that this system was not optimal, but given the local conditions, this system was the best compromise between available personnel, resources and compliance.

### Cycle 5: establishing an emergency room

The patients defined “red” by the simple triage system needed to be transported to a dedicated area or room for emergency treatment. The plan was to locate, brush up and equip a room suitable for patients in need of immediately care. A room that had previously been an emergency room was located. The room at this time was used for storage and garbage. Nurse students, staff and NGOs cleared, cleaned and painted the room. Four patient beds were found and renovated. Also, one oxygen cylinder that had been tucked away was repaired and filled. The one AMO and two nurses were permanently in the ER while the COs transferred their patients to the ER and continued the care themselves or handed the patient over to the AMO. To show progress, we decided to start admitting patients before the room was fully equipped. The new system of patients’ charts indicated which equipment and medications were most frequently used or needed. Based on the past weeks’ registration, we discussed the priority for further procurements at staff meetings. With minimal cost, it was possible to provide for basic emergency medical needs within a couple of months. Table 3 shows the medical equipment and medications available at the end of the project period.

**Table 3:** The equipment and medications available in the emergency room

• 4 beds
• 4 high-flow oxygen systems with disposable masks with reservoir
• Bag-valve-mask equipment (adult and paediatric)
• 1 manual suction device
• 2 pulse oximeters
• 4 stethoscopes
• 2 automatic blood pressure devices
• Disposables (syringes, needles, bandage, tape)
• Basic antibiotics
• Ketamine
• Benzodiazepine
• IV fluid bags (saline and glucose)
• Disposable nebulizers with beta stimulants
• 2 blood glucose devices
• Suturing equipment
• Pen and paper
• Registration forms
• Table and chairs
• Sterile gloves

## Discussion

We have described a small quality improvement project in a poorly resourced, overcrowded and underfunded emergency department in Zanzibar. The changes were achieved with minimal external funding. This was achieved by focusing on simple but essential changes that the indigenous personnel felt would represent an improvement of the system. The improvement method used was the Plan-Do-Study-Act (PDSA) rapid cycle method. This was easy to understand for the involved health care workers. The changes were organised into five themes, and each area of improvement followed the agreed structure. In addition, the bilateral exchange of experience between the local staff and the visitors from Norway and England was important to understand the local situation and challenges. In the following sections, we will discuss in more details some of the key elements that we think were major contributors to the process.

### Local ownership and leadership

The invitation to the project came from the hospital CEO. The head of department also took a leading role and was the main source of inspiration for the staff. The local head of department was the formal leader of the project with OT as his assistant. Without this local enthusiasm, the changes would not have been possible. Every step was discussed with the staff before the next intervention, and despite disagreement on details, the main goal was always a common cause. OT was present for the entire period, and GB, CM and his team were present for some weeks.

### System perspective and “change agents”

Fatigue and resignation due to a high number of self-referred patients characterized the staff before the project started. This bypass of primary health care is described in other African and European countries [6]. The first step was to introduce regular staff meetings, and this simple change enabled the staff to express their frustrations. More importantly, it also provided an opportunity for venting new ideas in a formal setting with their superior and the visiting doctors. Simple actions such as staff meetings do not pose yet another burden on a system close to collapse. The clear formulation of the aims of every intervention and continuous feedback on results updated on wall posters and at regular staff meetings enabled the staff to follow the process and monitor achievements. Local “change agents” or “champions” (particularly motivated and well-recognized staff) were identified and further encouraged. One or more of these individuals were present most days, passing their enthusiasm to their colleagues. The well-known phenomenon of “resistance to change” was less of a challenge in Zanzibar than in the visiting doctors’ own European hospitals [7]. Change was wanted, and the small improvements achieved then reinforced the willingness for further change. We believe that the focus on the long-term goal gave a long-lasting effect, as 1 year after the visiting doctors left, the system changes are still in place and even expanded.

### External funding

Donated medical equipment and consumables that were not essential to the plan were either sold or stored. Success in quality improvement is often more a matter of will, enthusiasm and organizational competence than of available financial resources [8]. From the start of the project, it was made clear that the visiting doctors did not bring any extra funding or equipment. This assumption prevented any local expectations that new and expensive equipment could solve their problems easily. Later in the project, some equipment was nevertheless acquired from external sources. These were low-maintenance, high-use equipment such as sphygmomanometers, auroscopes and otoscopes (all wall mounted), peak flow meters and a pulse oximeter. Developing and relying on local shops for equipment and medications should be an aim of north-south cooperation, like our project.

### Further challenges

Change and improvement in just one part of the patients’ hospital journey does not change the entire system. The patient relies on well-organized cooperation with other departments and specialists after the initial treatment in the ED. Lack of official and formal meetings across departments complicates patient flow. A focus on the entire chain of survival is not only a matter of optimal utilization of resources and funding. Equally important is willingness to change and the knowledge of simple quality improvement methods, in order to improve care processes. At the end of the project, other specialties became involved in developing treatments and procedures in the OPD. This interdisciplinary cooperation represents the first step in making the ED the main door into the hospital.

## Conclusions

Change and improvement in health care are achievable despite limited financial resources if a comprehensive, robust and simple system is used and adhered to even if the day-to-day changes are small. Involvement of all stakeholders from the start, identification and use of change agents, regular feedback and a focus on human resources rather than equipment have been key factors for the success of this project.

### Ethics

The project was approved by the Ethic Committee at Mnazi Mmoja Hospital.

## Abbreviations

AMO: assistant medical officer
COs: clinical officers
ED: emergency department
HUH: Haukeland University Hospital
MMH: Mnazi Mmoja Hospital
OPD: outpatient department
PDSA: Plan-Do-Study-Act
